# Astaxanthin Inhibits Oxidative Stress-Induced Ku Protein Degradation and Apoptosis in Gastric Epithelial Cells

**DOI:** 10.3390/nu14193939

**Published:** 2022-09-22

**Authors:** Jaeeun Lee, Joo Weon Lim, Hyeyoung Kim

**Affiliations:** Department of Food and Nutrition, BK21 FOUR, College of Human Ecology, Yonsei University, Seoul 03722, Korea

**Keywords:** apoptosis, astaxanthin, gastric epithelial cells, Ku proteins, oxidative stress

## Abstract

Oxidative stress induces DNA damage which can be repaired by DNA repair proteins, such as Ku70/80. Excess reactive oxygen species (ROS) stimulate the activation of caspase-3, which degrades Ku 70/80. Cells with decreased Ku protein levels undergo apoptosis. Astaxanthin exerts antioxidant activity by inducing the expression of catalase, an antioxidant enzyme, in gastric epithelial cells. Therefore, astaxanthin may inhibit oxidative stress-induced DNA damage by preventing Ku protein degradation and thereby suppressing apoptosis. Ku proteins can be degraded via ubiquitination and neddylation which adds ubiquitin-like protein to substrate proteins. We aimed to determine whether oxidative stress decreases Ku70/80 expression through the ubiquitin–proteasome pathway to induce apoptosis and whether astaxanthin inhibits oxidative stress-induced changes in gastric epithelial AGS cells. We induced oxidative stress caused by the treatment of β-D-glucose (G) and glucose oxidase (GO) in the cells. As a result, the G/GO treatment increased ROS levels, decreased nuclear Ku protein levels and Ku-DNA-binding activity, and induced the ubiquitination of Ku80. G/GO increased the DNA damage marker levels (γ-H2AX; DNA fragmentation) and apoptosis marker annexin V-positive cells and cell death. Astaxanthin inhibited G/GO-induced alterations, including Ku degradation in AGS cells. MLN4924, a neddylation inhibitor, and MG132, a proteasome inhibitor, suppressed G/GO-mediated DNA fragmentation and decreased cell viability. These results indicated that G/GO-induced oxidative stress causes Ku protein loss through the ubiquitin–proteasome pathway, resulting in DNA fragmentation and apoptotic cell death. Astaxanthin inhibited oxidative stress-mediated apoptosis via the reduction of ROS levels and inhibition of Ku protein degradation. In conclusion, dietary astaxanthin supplementation or astaxanthin-rich food consumption may be effective for preventing or delaying oxidative stress-mediated cell damage by suppressing Ku protein loss and apoptosis in gastric epithelial cells.

## 1. Introduction

Oxidative stress oxidizes nucleotides and causes DNA damage [1,2]. Carcinogenesis is induced by genomic instability due to DNA damage [3]. The damaged DNA can be repaired by various pathways including via base adductions, deletions, nucleotide excisions, and other pathways [4,5,6]. The two main repair pathways are the homologous recombination (HR) and non-homologous end-joining (NHEJ) pathways [7]. NHEJ involves Ku70/80 proteins and is susceptible to chromosomal rearrangements [8,9]. 

Regarding oxidative stress and Ku protein degradation, we previously showed that excess amounts of reactive oxygen species (ROS) increase caspase-3 activity and decrease nuclear Ku proteins, Ku-DNA binding activity, and cell viability in gastric epithelial AGS cells treated with β-carotene [10]. Oxidative stress induced by the treatment of β-D-glucose (G) and glucose oxidase (GO), dramatically decreases nuclear Ku protein levels [11]. We previously showed that oxidative stress activated caspase-3, which degrades Ku 70/80 in pancreatic acinar cells [11]. G/GO treatment increases the activity of calpain and caspase-3 and induces apoptosis. It decreases cell viability and increases the Bax/BCl-2 ratio and DNA fragmentation in pancreatic acinar cells [12]. G/GO increases the loss of the DNA repair protein ataxia telangiectasia-mutated (ATM), which is involved in HR pathways. Son et al. [13] showed that the exposure of cells to hydrogen peroxide produced by G/GO treatment causes caspase-independent and apoptosis-inducing factor (AIF)-dependent cell death in B and T lymphoma cells. In addition, G/GO induces caspase-3 activation and apoptotic cell death, which is followed by cytochrome c release from the mitochondria in glial cells [14]. These studies suggested that oxidative stress degrades DNA repair proteins via caspase-dependent or AIF-dependent pathways. Recently, we demonstrated that excessive amounts of ROS induce caspase-3 cleavage and truncation of AIF with a loss of Ku proteins, leading to apoptosis in *Helicobacter pylori (H. pylori)*-infected cells [15]. Therefore, the mechanism of degradation of DNA repair Ku proteins should be investigated to prevent persistent DNA damage-induced genomic instability and carcinogenesis.

Ku proteins, heterodimers consisting of Ku70 and Ku80 subunits, are major players in the NHEJ repair pathways [16]. NHEJ, the major DNA repair system in mammalian cells, is important for genomic stability and malignant transformation suppression and requires Ku, DNA-dependent protein kinase catalytic subunit (DNA-PKcs), X-ray repair cross-complementing protein 4 (XRCC4), DNA ligase 4, and XRCC4-like factor (XLF) [17]. Ku proteins bind to the broken DNA ends and recruit DNA-PKcs to the DNA double-strand break (DSB) site. During DSBs, the phosphorylation of H2A histone family member X (H2AX) occurs and forms γ-H2AX [18]. Ser-139-phosphorylated γ-H2AX is an established DSB marker, and γ-H2AX foci formation is used as an indicator of DNA damage [18]. We previously demonstrated that *H. pylori*-induced oxidative stress led to γ-H2AX foci formation, a decrease in Ku proteins, and an increase in ROS levels in gastric epithelial cells [15]. Therefore, antioxidants may inhibit ROS-induced Ku protein loss and oxidative stress-mediated apoptosis.

The ubiquitin–proteasome system is important for the degradation of proteins in mammalian cells [19]. It involves the attachment of the small protein ubiquitin to the target proteins. This event modifies the properties of proteins. Additional ubiquitin molecules are then conjugated and form a polyubiquitinated adduct of proteins. This polyubiquitinated adduct is recognized by the 26S proteasome and the substrate proteins are degraded. Postow et al. [20] demonstrated that Ku proteins are attached to DNA during the DNA repair process. After repairing DSBs, Ku80 efficiently eliminates Ku from DNA through the ubiquitination of Ku80 [20]. Therefore, it is important to determine whether the oxidative stress-induced loss of Ku proteins is caused by the ubiquitination/proteasomal degradation of Ku proteins.

In addition to the ubiquitin–proteasome pathway, neddylation mediates Ku degradation by promoting ubiquitination and Ku release from the DNA damage sites [21]. Neddylation is a reversible post-translational modification of proteins. It adds a ubiquitin-like protein called ‘neuronal precursor cell-expressed developmentally downregulated protein 8’ (NEDD8) to the substrate proteins [22]. MLN4924 is a NEDD8-activating enzyme inhibitor. It specifically inhibits the conjugation of NEDD8 to the target proteins [23]. MLN492 inhibits ubiquitination and Ku protein loss, suggesting that neddylation promotes Ku protein ubiquitination [21]. Therefore, Ku protein neddylation is associated with Ku protein ubiquitination, which may induce apoptotic cell death.

Astaxanthin, an oxygen-containing carotenoid (xanthophyll), is present in marine algae, red yeast, fungi, and various plant and animal sources. It is a fat-soluble nutrient and one of the most potent antioxidants owing to its molecular structure and arrangement in the plasma membrane [24,25]. Astaxanthin reduces ROS levels by inducing the ‘peroxisome proliferator-activated receptor-γ’ (PPAR-γ) target gene catalase in gastric epithelial cells [26].

The present study aimed to determine whether G/GO-generated oxidative stress decreases Ku70/80 levels via the ubiquitin–proteasome pathway to induce apoptosis and whether astaxanthin inhibits G/GO-mediated Ku70/80 loss, DNA damage, and apoptosis by reducing ROS levels in gastric epithelial AGS cells.

To determine the involvement of ubiquitination/proteasomal degradation and neddylation, MLN4924, a neddylation inhibitor (a NEDD8-activating enzyme inhibitor), and MG132, a proteasome inhibitor, were treated in the cells prior to the treatment of G/GO.

## 2. Materials and Methods

### 2.1. Cell Culture

The human gastric adenocarcinoma cell line AGS (ATCC CRL 1739) was purchased from the American Type Culture Collection (Rockville, MD, USA) and grown in RPMI-1640 medium (GIBCO; Grand Island, NY, USA) supplemented with 10% fetal bovine serum (GIBCO), 2 mM glutamine, 100 U/mL penicillin, and 100 μg/mL streptomycin (Sigma-Aldrich Corp., St. Louis, MO, USA) at 37 °C in a humidified atmosphere containing 95% air and 5% CO_2_.

### 2.2. Experimental Protocol

Astaxanthin (Sigma-Aldrich) dissolved in dimethyl sulfoxide (DMSO) was stored in nitrogen at −80 °C, and the cells were treated with astaxanthin (1, 2, or 5 µM) for 3 h, followed by stimulation with G (10 mM)/GO (5 mU/mL) for 30 min (to measure intracellular ROS levels), 8 h (to determine Ku levels, Ku–DNA binding activity, and Ku ubiquitination), and 16 h (to assess cell viability, apoptosis marker annexin V-stained cells, and DNA damage marker levels, i.e., γ-H2AX and DNA fragmentation).

To determine the mechanism of Ku degradation, the cells were pre-treated with MLN4924 and MG132 (dissolved in DMSO and diluted in RPMI 1640 to 5 nM and 0.5 µM, respectively) for 1 h, followed by stimulation with G/GO for 16 h. Control cells without astaxanthin, MLN4924, or MG-132 treatment were incubated with less than 0.1% DMSO.

### 2.3. Intracellular ROS Level Measurement

The cells (2.0 × 10^5^/2 mL/well) were incubated with 10 µM dichlorofluorescein diacetate (DCF-DA; Sigma-Aldrich) in 5% CO_2_/95% air at 37 °C. Intracellular ROS levels were measured by the method previously described [26]. The ROS level of the untreated cells was set to 100%.

### 2.4. Western Blot Analysis

Preparation of whole-cell and nuclear extracts and Western blot analysis were performed as previously described [26]. Specific antibodies against Ku70 (sc-5309; dilution 1/3000), Ku80 (sc-5280; dilution 1/3000), actin (sc-1615; dilution 1/4000) (all from Santa Cruz Biotechnology, Dallas, TX, USA), and lamin B1 (ab-16048, Abcam; Cambridge, UK; dilution 1/2000) were used. After incubation with secondary antibodies (anti-goat, -mouse, or -rabbit antibodies conjugated to horseradish peroxidase; Santa Cruz Biotechnology), the proteins were visualized using a Clarity Western ECL Substrate (705061; Bio-Rad) and a ChemiDoc imaging system (Bio-Rad Laboratories Inc.). The protein levels of Ku80 and Ku70 in the whole-cell or nuclear extracts were compared to those of the loading control actin or lamin B1, respectively. The intensity of each protein band was densitometrically quantified using the ImageJ software (National Institutes of Health, Bethesda, MD, USA). Densitometry data represent the mean ± standard error (SE) from three immunoblots. For relative protein expression, the relative density of the protein band was normalized to actin or laminB1.

### 2.5. Electrophoretic Mobility Shift Assay (EMSA)

Ku–DNA binding activity was determined using EMSA, followed by the method described previously [15] using a ^32^P-labeled double-stranded oligonucleotide 5′-GGGCCAAGAATCTTAGCAGTTTCGGG-3′. Quantification of the Ku complexes was performed by densitometry. The intensity of the Ku band was densitometrically quantified using the ImageJ software (National Institutes of Health, Bethesda, MD, USA). The densitometry data represent the mean ± SE from the three Ku bands.

### 2.6. Cell Viability and DNA Fragmentation Determination

The cells (5.0 × 10^4^/mL/well) were cultured with or without treatment. Cell viability and DNA fragmentation were determined as previously described [15]. The number of viable untreated cells was set to 100%. DNA fragmentation expressed as an enrichment factor of untreated cells was set to 1.

### 2.7. Annexin V/Propidium Iodide (PI)—Staining Assay

Apoptosis was measured by flow cytometry using an annexin V–fluorescein isothiocyanate (FITC)/PI staining kit (BD Biosciences, San Jose, CA, USA). The cells (1.0 × 10^6^/10 mL/dish) were collected, washed with PBS, and resuspended in 200 μL of 1× binding buffer containing annexin V (1:50 according to the manufacturer’s instructions) and 20 ng/sample PI for 15 min at 21–23 °C in the dark. Viable, apoptotic, and necrotic cell counts were quantified using flow cytometry (Becton Dickinson; Franklin Lakes, NJ, USA) and analyzed using CellQuest software. The cells were excited at 488 nm, and the emissions of annexin V at 525 nm and PI were collected using 610 nm band-pass filters. A total of 10,000 cells were analyzed for each sample. Apoptotic cells were expressed as a percentage of total cells ([apoptotic cell count]/[total observed cell count] × 100). Apoptotic cells included annexin V^+^/PI^−^ cells (early apoptosis) and annexin V^+^/PI^+^ cells (late apoptosis).

### 2.8. Immunofluorescence Staining for γ-H2AX

Immunofluorescence staining for γ-H2AX and 4,6-diamidino-2-phenylindole (DAPI) was determined as previously described [15] using a confocal laser-scanning microscope (Zeiss LSM 880, Carl Zeiss Inc., Thornwood, NY, USA). Nuclear γ-H2AX fluorescence was determined using the ZEN Blue 3.1 software (Carl Zeiss Inc., Thornwood, NY, USA).

### 2.9. Co-Immunoprecipitation Assay

The cells (1.0 × 10^6^/10 mL) were lysed in an immunoprecipitation buffer, and the co-immunoprecipitation assay was determined as previously described [15] using an anti-ubiquitin antibody (sc-8017) and protein A/G PLUS-agarose (sc-2003; both from Santa Cruz Biotechnology). Protein G-antibody-antigen complexes were collected. Ku80 expression was detected using Western blotting. Whole-cell extracts that were not incubated with the antibody and protein G/A were also subjected to Western blot analysis to detect the expression of Ku80 and actin as input proteins. The quantification of ubiquitin binding to Ku80 was based on the band intensity of the Western blot analysis using the Image J software. Densitometric data represent the mean ± SE from three immunoblots.

### 2.10. Statistical Analysis

All values are expressed as the mean ± SE of three independent experiments. For each experiment, four samples were placed in each group (the total number in each group was 12). Analysis of variance (ANOVA) followed by Tukey’s post hoc test were used for statistical analysis. Differences were considered statistically significant at *p* < 0.05.

## 3. Results

### 3.1. G/GO Induces Cell Death, DNA Fragmentation, and Apoptosis in AGS Cells

**Figure 1:**
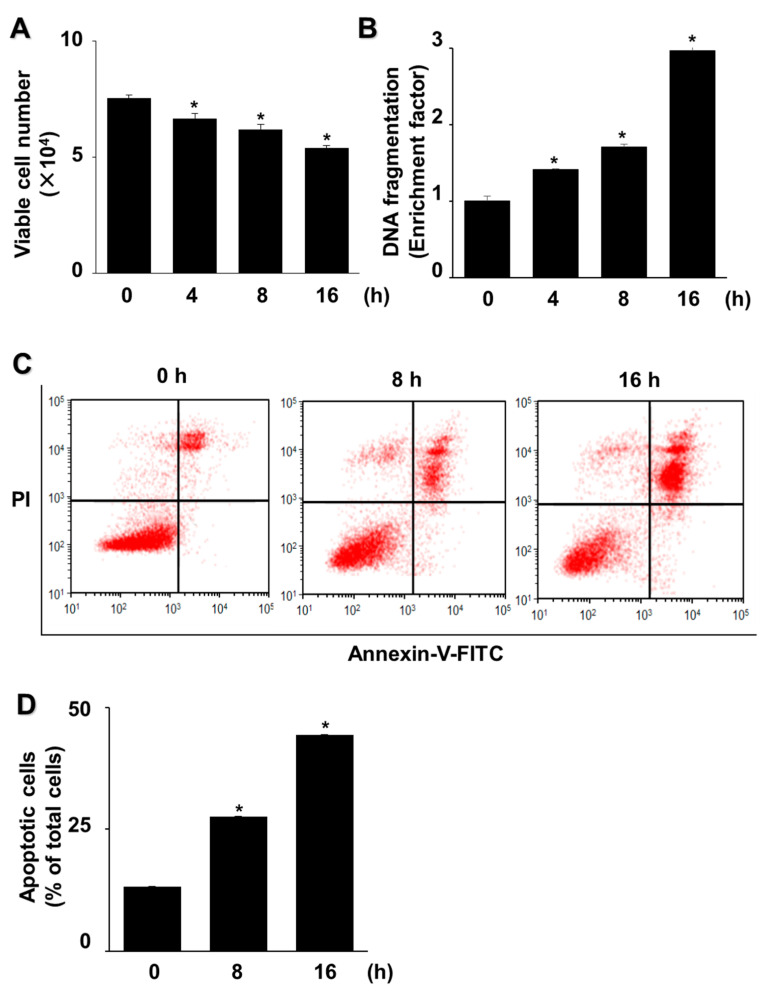
Glucose/glucose oxidase (G/GO) treatment induced cell death, DNA fragmentation, and apoptosis in AGS cells. The cells were stimulated with G/GO for the indicated periods. (**A**) Viable cell counts were determined using the trypan blue exclusion test. (**B**) DNA fragmentation, expressed as an enrichment factor of the untreated cells (the cells at 0 h), was set at 1. (**C**) Apoptosis was measured by flow cytometry using annexin V−fluorescein isothiocyanate (FITC)/propidium iodide (PI) staining. (**D**) A bar graph of FITC−labeled annexin V/PI staining is shown. Apoptotic cells were expressed as % of total cells ([apoptotic cell count]/[total observed cell count] × 100). All data are shown as mean ± standard error (SE) of three independent experiments. For each experiment, four samples were placed in each group (the total number of each group was 12). * *p* < 0.05 vs. untreated cells (the cells at 0 h).

AGS cells exposed to G/GO for 4, 8, or 16 h showed reduced viability (Figure 1A). Similarly, the G/GO treatment increased DNA fragmentation in a time-dependent manner (Figure 1B). To further assess G/GO-induced apoptosis, we examined phosphatidylserine exposure on the cell surface using annexin V/PI double staining. Flow cytometry analysis indicated that the percentage of annexin V^+^ apoptotic cells increased in a time-dependent manner until 16 h (Figure 1C,D). Therefore, a 16 h culture was used to determine the effect of astaxanthin on the G/GO-induced decrease in cell viability and apoptosis.

### 3.2. Astaxanthin Inhibits G/GO-Induced Cell Death, DNA Fragmentation, and Apoptosis in AGS Cells

**Figure 2:**
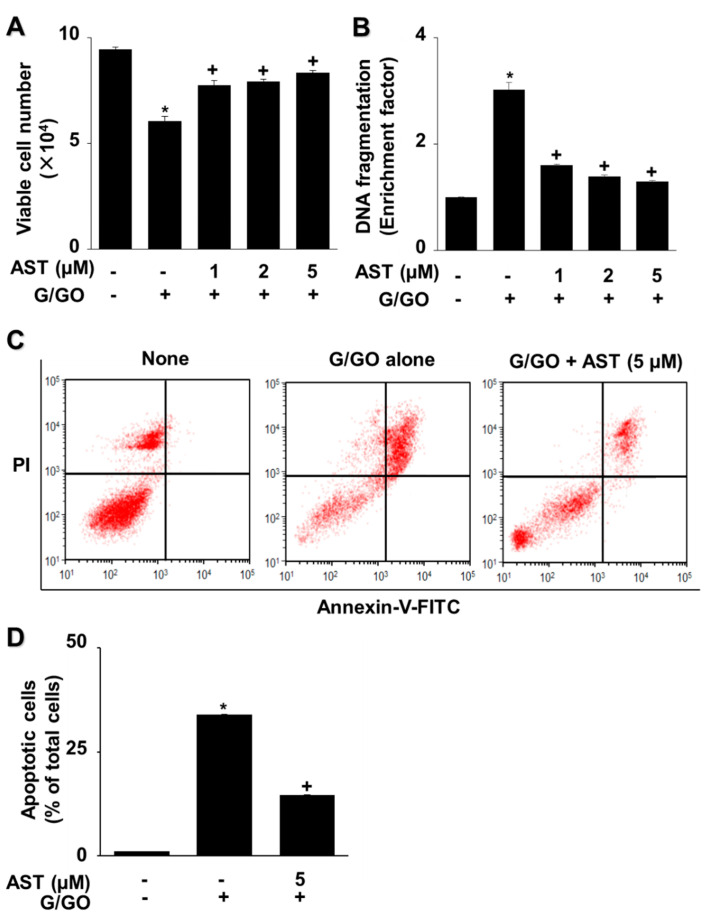
Astaxanthin increases cell viability and decreases DNA fragmentation and apoptosis in glucose/glucose oxidase (G/GO)−stimulated AGS cells. The cells were treated with the indicated astaxanthin concentrations for 3 h, followed by G/GO treatment for 16 h. (**A**) Viable cell counts were determined using the trypan blue exclusion test. (**B**) DNA fragmentation expressed as an enrichment factor of the untreated cells (AST− and G/GO−: cells without astaxanthin treatment and in the absence of G/GO) was set at 1. (**C**) Apoptosis was measured by flow cytometry using annexin V−fluorescein isothiocyanate (FITC)/propidium iodide (PI) staining. (**D**) A bar graph of FITC−labeled annexin V/PI staining is shown. Apoptotic cells were expressed as % of total cells ([apoptotic cell count]/[total observed cell count] × 100). All data are shown as mean ± standard error (SE) of three independent experiments. For each experiment, four samples were placed in each group (the total number in each group was 12). * *p* < 0.05 vs. untreated cells (AST− and G/GO−). + *p* < 0.05 vs. control cells (AST− and G/GO+: cells without astaxanthin treatment in the presence of G/GO). AST, astaxanthin.

Astaxanthin inhibited the G/GO-induced decrease in viable cell count after culturing for 16 h and suppressed G/GO-induced DNA fragmentation in a dose-dependent manner (Figure 2A,B). In addition, astaxanthin inhibited the percentage of Annexin V^+^ apoptotic cells in G/GO-treated cells (Figure 2C,D). These data show that astaxanthin inhibits G/GO-induced cell death, DNA fragmentation, and apoptosis in AGS cells in a dose-dependent manner.

### 3.3. Astaxanthin Inhibits G/GO-Induced Increase in ROS Levels and γ-H2AX in AGS Cells

**Figure 3:**
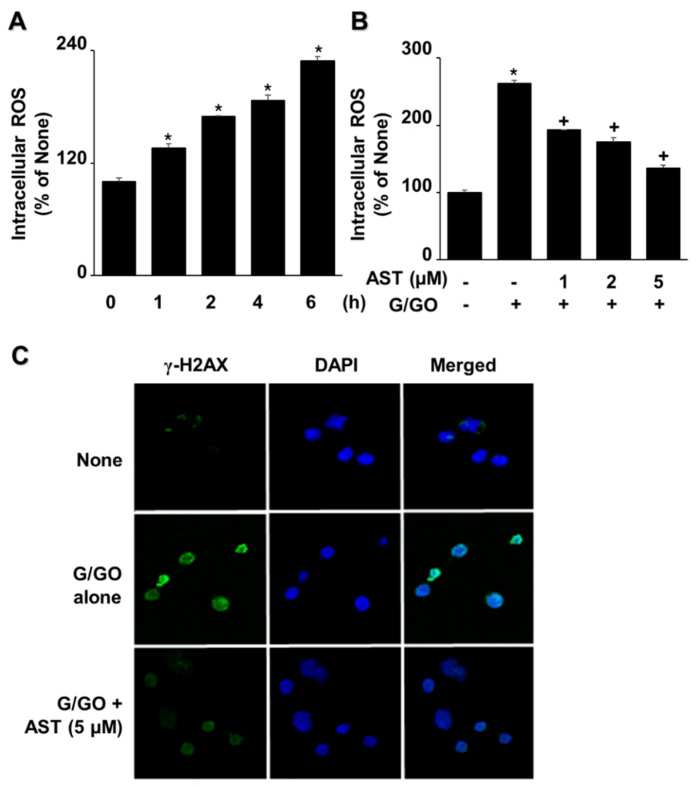
Astaxanthin inhibited the glucose/glucose oxidase (G/GO)−induced increase in reactive oxygen species (ROS) levels and γ-H2AX foci formation in AGS cells. (**A**) The cells were treated with G/GO for the indicated time periods. (**B**) The cells were pre-treated with the indicated concentrations of astaxanthin for 3 h and subsequently stimulated with G/GO for 6 h. (**A**,**B**) Intracellular ROS levels were determined using dichlorofluorescein (DCF) fluorescence. The value for ROS levels in untreated cells (AST− and G/GO−: cells without astaxanthin treatment, in the absence of G/GO) was set at 100%. All data are shown as mean ± standard error (SE) of three independent experiments. For each experiment, four samples were placed in each group (the total number in each group was 12). * *p* < 0.05 vs. untreated cells (AST− and G/GO−). + *p* < 0.05 vs. control cells (AST− and G/GO+: cells without astaxanthin treatment in the presence of G/GO). (**C**) The cells were treated with 5 μM astaxanthin for 3 h, followed by G/GO treatment for 16 h. Cells and nuclei were stained with anti-γ-H2AX antibody (green) and DAPI (blue), respectively. AST, astaxanthin.

G/GO increased intracellular ROS levels in AGS cells in a time-dependent manner for 6 h (Figure 3A). However, astaxanthin decreased intracellular ROS levels in G/GO-treated cells after 6 h in a dose-dependent manner (Figure 3B). The G/GO treatment significantly increased robust γ-H2AX foci formation (Figure 3C, panel 2), which was inhibited by astaxanthin (Figure 3C, panel 3). These results suggest that astaxanthin reduces ROS levels and inhibits DNA damage in G/GO-treated cells.

### 3.4. Astaxanthin Inhibits G/GO-Induced Ku70/80 Loss in AGS Cells

**Figure 4:**
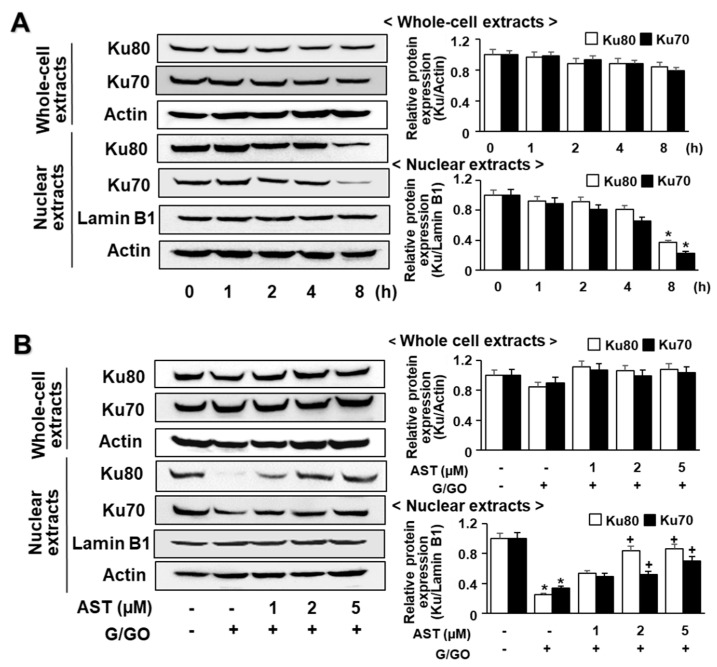
Astaxanthin inhibits glucose/glucose oxidase (G/GO)−induced decrease in Ku70/80 levels in whole-cell extracts and nuclear extracts in AGS cells. The cells were (**A**) treated with G/GO for the indicated time periods or (**B**) pre−treated with the indicated concentrations of astaxanthin for 3 h, followed by stimulation with G/GO for 8 h. (**A**,**B**) Ku70 and 80 protein levels in the whole−cell and nuclear extracts were determined by Western blot analysis using actin and lamin B1 as the loading control and nuclear marker, respectively (left panel). The densitometry data represent mean ± standard error (SE) from three immunoblots and are shown as the relative density of the actin or lamin B1 protein bands (right panel). * *p* < 0.05 vs. untreated cells (AST− and G/GO−). + *p* < 0.05 vs. control cells (AST− and G/GO+: cells without astaxanthin treatment in the presence of G/GO). AST, astaxanthin.

**Figure 5:**
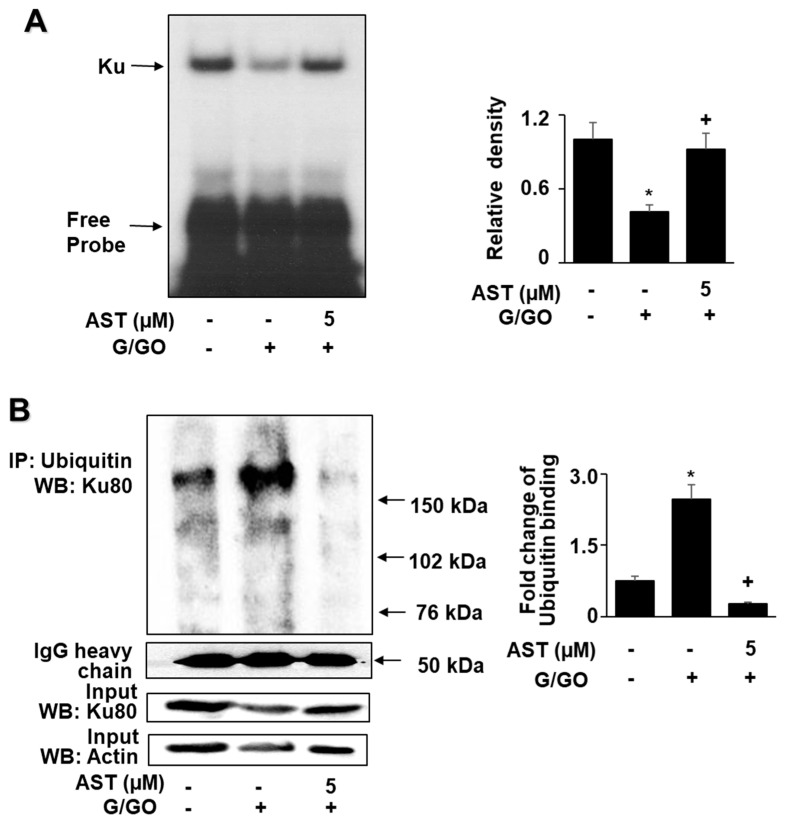
Astaxanthin inhibits glucose/glucose oxidase (G/GO) −induced decrease in Ku−DNA binding activity and Ku80 ubiquitination in AGS cells. The cells were pre-treated with 5 μM of astaxanthin for 3 h, followed by stimulation with G/GO for 8 h. (**A**) The Ku–DNA binding activity was determined using electrophoretic mobility shift assay (EMSA) (left panel). The quantification of the Ku–DNA binding activity was performed using densitometric analysis. Bar graph showing the relative density of the EMSA autoradiograph of the three groups compared to the untreated group (right panel). (**B**) Whole−cell extracts were immunoprecipitated (IP) with the anti-ubiquitin antibody, followed by Western blot analysis with the anti−Ku80 antibody. The input protein, Ku80, and actin levels were determined by Western blot analysis. The input was used as a control for protein expression. The IgG heavy chain was shown to have a molecular weight of 50 kDa (left panel). The quantification of the binding of ubiquitin to Ku80 was performed based on the band intensity of the Western blot analysis using Image J software (right panel). The densitometry data represent mean ± standard error (SE) from three immunoblots. * *p* < 0.05 vs. untreated cells (AST− and G/GO−). + *p* < 0.05 vs. control cells (AST− and G/GO+: cells without astaxanthin treatment in the presence of G/GO). AST, astaxanthin.

G/GO decreased the Ku levels in the nuclear extracts after culturing for 8 h (Figure 4A). Ku proteins in the whole-cell extracts were not significantly altered by the G/GO treatment. Astaxanthin significantly inhibited the G/GO-induced decrease in Ku70/80 in the nuclear extracts (Figure 4B). To determine the effect of astaxanthin on the Ku–DNA binding activity in the G/GO-stimulated AGS cells, the nuclear extracts were incubated with ^32^P-labeled Ku oligonucleotides, resolved by electrophoresis, and exposed to radiography film. Treatment with G/GO for 8 h reduced the Ku–DNA binding activity, which was significantly inhibited by astaxanthin (Figure 5A).

To investigate whether Ku80 ubiquitination was involved in G/GO-induced Ku80 loss, we used co-immunoprecipitation combined with Western blotting to detect ubiquitinated Ku80. The G/GO treatment increased the levels of ubiquitin-associated Ku80 (Figure 5B), indicating that Ku80 ubiquitination was involved in G/GO-induced Ku80 loss. As one or more high-molecular-weight bands were observed when stained with the Ku80 antibody, Ku80 was subjected to polyubiquitination. The typical ubiquitinated Ku80 protein is >150 kDa. Astaxanthin (5 µM) inhibited the G/GO-induced increase in ubiquitinated Ku80 (Figure 5B). These results suggested that astaxanthin inhibited G/GO-induced Ku80 ubiquitination in AGS cells. The input protein, Ku80, and actin levels were not affected by the treatment. The input was used as a control for protein expression. The IgG heavy chain was shown to have a molecular weight of 50 kDa and was not changed by the treatment.

### 3.5. MLN4924 and MG132 Inhibit G/GO-Induced Cell Death, DNA Fragmentation, and Apoptosis in AGS Cells

**Figure 6:**
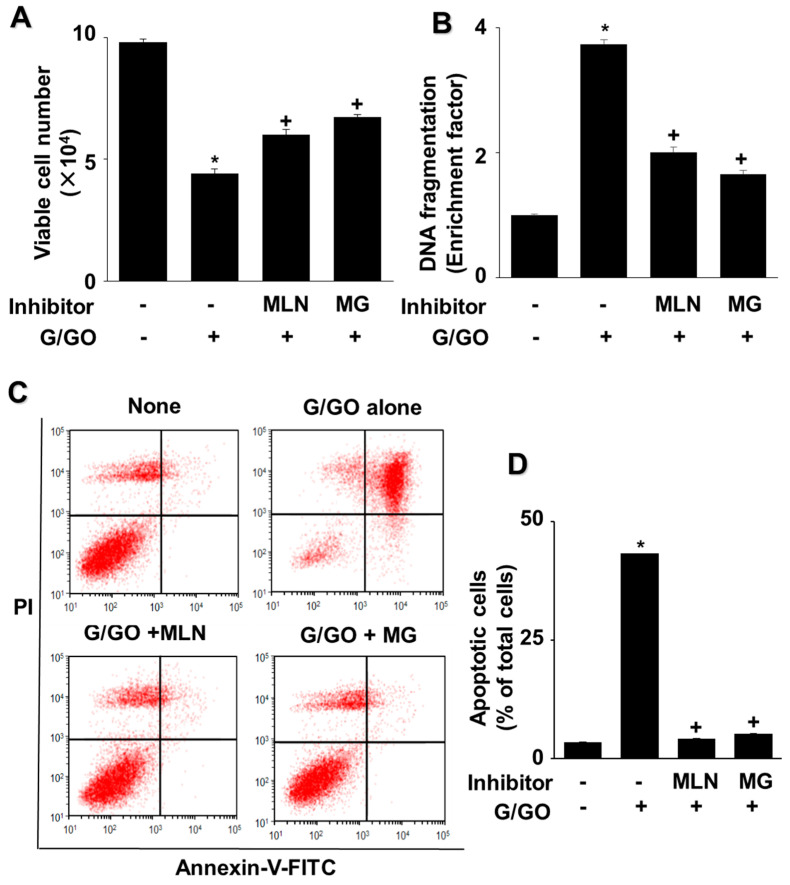
MLN4924 and MG132 inhibited glucose/glucose oxidase (G/GO)−induced cell death, DNA fragmentation, and apoptosis. The cells were incubated with 5 nM MLN4924 or 0.5 µM MG132 for 1 h, then stimulated with G/GO for 16 h. (**A**) Viable cell counts were determined using the trypan blue exclusion test. (**B**) DNA fragmentation, expressed as an enrichment factor of untreated cells (AST− and G/GO−: cells without astaxanthin treatment, in the absence of G/GO) was set at 1. (**C**) Apoptosis was measured by flow cytometry using annexin V−fluorescein isothiocyanate (FITC)/propidium iodide (PI) staining. (**D**) A bar graph of FITC−labeled annexin V/PI staining is shown. Apoptotic cells were expressed as % of total cells ([apoptotic cell count]/[total observed cell count] × 100). All data are shown as mean ± standard error (SE) of three independent experiments. * *p* < 0.05 vs. untreated cells (AST− and G/GO−: cells without astaxanthin treatment in the absence of G/GO). + *p* < 0.05 vs. control cells (Inhibitor− and G/GO+: cells without MLN4924 or MG132 treatment in the presence of G/GO). MLN, MLN4924; MG, MG132.

MG132 and MLN4924 inhibited the G/GO-induced decrease in the number of viable cells (Figure 6A). In addition, MLN4924 and MG132 significantly suppressed the G/GO-induced increase in DNA fragmentation (Figure 6B) and apoptosis, as assessed by the percentage of annexin V^+^ apoptotic cells (Figure 6C,D) in the AGS cells. These results indicate that G/GO-induced cell death and DNA fragmentation are associated with ubiquitin–proteasome-mediated Ku protein proteolysis in AGS cells.

## 4. Discussion

This study shows that oxidative stress generated by G/GO causes oxidative DNA damage, determined by γ-H2AX and DNA fragmentation, and decreases nuclear Ku proteins and Ku–DNA binding activity but increases the ubiquitination of Ku80, apoptosis marker annexin V-stained cells, and cell death in AGS cells. However, MLN4924 and MG132 inhibit DNA fragmentation and apoptosis in the G/GO-treated AGS cells. The results show that oxidative stress degrades Ku70/80 through the ubiquitin–proteasome pathway, thereby causing the nuclear loss of Ku70/80 in AGS cells. Astaxanthin decreases ROS levels and suppresses ubiquitin–proteasome-mediated Ku70/80 degradation, Ku protein loss, DNA damage, and apoptosis in G/GO-stimulated AGS cells. Therefore, astaxanthin-rich food consumption may be effective for preventing or delaying ROS-induced cell damage by suppressing Ku70/80 loss and apoptotic cell death in gastric epithelial cells.

Oxidative stress is an important pathogenic factor in the development of inflammatory diseases and cancer. Astaxanthin exerts antioxidant effects in preventing oxidative stress-related diseases by scavenging free radicals. Many studies have shown that astaxanthin reduces ROS levels and inhibits cellular apoptosis by enhancing the activity of antioxidant enzymes including glutathione peroxidase and catalase in different cell lines [27,28,29].

Excessive ROS downregulate Ku70/80 and NHEJ proteins and apoptotic cell death [10,11,30,31]. Ku is a dimeric protein complex that maintains genomic integrity [32]. Regarding Ku protein degradation, neddylation at DSB sites promotes the ubiquitination of Ku after DNA damage [21]. The ubiquitin–proteasome pathway is the main intracellular and non-lysosomal protein degradation system and regulates several cellular processes including gene expression control and cell cycle progression [33]. After the repair process, Ku80 is ubiquitinated and removed from the DNA [20]. Therefore, the ubiquitin–proteasome pathway may be involved in the oxidative stress-induced degradation of Ku proteins. The present study shows that both MLN4924 and MG132 inhibit oxidative stress-mediated apoptotic cell death and fragmentation of DNA in AGS cells, suggesting that oxidative stress induces ubiquitin–proteasome-mediated Ku protein degradation and thus apoptosis in AGS cells. The present results are supported by a previous finding showing that oxidative stress induces apoptosis and decreases Ku–DNA binding activity in pancreatic acinar cells [34]. 

The present study shows that oxidative stress caused by G/GO does not change Ku proteins in whole-cell extracts but dramatically decreases nuclear Ku proteins. We previously demonstrated that Ku70/80 in whole-cell extracts were not changed by the treatment of G/GO in pancreatic acinar cells [11]. The nuclear loss of Ku proteins caused by G/GO results from the Ku protein degradation and decreased Ku binding to nuclear transporter importins in pancreatic acinar cells. 

Astaxanthin reduces ROS by inducing antioxidant enzyme catalase, which inhibits IL-8 expression in *H. pylori*-stimulated gastric epithelial cells [26]. Astaxanthin also suppresses cell invasion and migration in *H. pylori*-stimulated gastric epithelial cells [35]. These studies suggest that the beneficial effects of astaxanthin on inflammatory cytokine expression and invasion are attributed to its antioxidant activity. 

Sudharshan and Dyavaiah reported that astaxanthin prevents ROS-induced apoptotic cell death with the fragmentation of DNA in yeast [36]. Astaxanthin, zeaxanthin, and lutein inhibit reactive nitrogen species-induced DNA fragmentation in neuroblastoma cells [37]. These carotenoids protect against DNA damage in UVA-exposed rat epithelial cells [38]. In human lymphocytes, astaxanthin reduces radiation-induced chromosomal aberrations and DNA damage [39]. These studies support the present finding that astaxanthin inhibits oxidative stress-induced Ku70/80 loss, thus preventing apoptotic cell death in gastric epithelial cells. 

## 5. Conclusions

Astaxanthin inhibits oxidative stress-induced apoptotic cell death via the reduction of ROS, the prevention of Ku protein loss through the ubiquitin–proteasome pathway, and the protection of DNA damage in gastric epithelial cells. Thus, astaxanthin supplementation or astaxanthin-rich food consumption may protect oxidative stress-induced gastric damage by preventing Ku protein loss and apoptosis in gastric epithelial cells. 

## Data Availability

All data generated or analyzed during this study are included in this published article.
